# Detection of three pandemic causing coronaviruses from non-respiratory samples: systematic review and meta-analysis

**DOI:** 10.1038/s41598-021-95329-4

**Published:** 2021-08-09

**Authors:** Chandan Mishra, Suneeta Meena, Jitendra Kumar Meena, Suman Tiwari, Purva Mathur

**Affiliations:** 1 Department of Laboratory Medicine, All India Institute of Medical Sciences, Delhi, India; 2 Preventive Oncology, NCI Jhajjar, All India Institute of Medical Sciences, Delhi, India; 3 Department of Anaesthesia and Intensive Care, VMMC and Safdarjung Hospital, Delhi, India; 4 Department of Laboratory Medicine, JPNATC, All India Institute of Medical Sciences, Delhi, India

**Keywords:** Microbiology, Medical research

## Abstract

SARS-CoV-2 has posed an unprecedented challenge to the world. Pandemics have been caused previously by viruses of this family like Middle East Respiratory Corona Virus (MERS CoV), Severe Acute Respiratory Syndrome Corona Virus (SARS CoV). Although these viruses are primarily respiratory viruses, but they have been isolated from non-respiratory samples as well. Presently, the detection rate of SARS‐CoV‐2 RNA from different clinical specimens using Real Time Reverse Transcriptase Polymerized Chain Reaction (qRT‐PCR) after onset of symptoms is not yet well established. Therefore, the aim of this systematic review was to establish the profile of detecting SARS‐CoV‐2, MERS CoV, SARS CoV from different types of clinical specimens other than the respiratory using a standard diagnostic test (qRT‐PCR). A total of 3429 non-respiratory specimens were recorded: SARS CoV (total sample—802), MERS CoV (total sample—155), SARS CoV-2 (total sample—2347). Out of all the samples studied high positive rate was seen for saliva with 96.7% (14/14; 95% CI 87.6–100.0%) for SARS CoV and 57.5% (58/250; 95% CI − 1.2 to 116.2%) for SARS CoV-2, while low detection rate in urine samples for SARS CoV-2 with 2.2% (8/318; 95% CI 0.6–3.7%) and 9.6% (12/61; 95% CI − 0.9 to 20.1%) for SARS CoV but there was relatively higher positivity in urine samples for MERS CoV with detection rate of 32.4% (2/38; 95% CI − 37.3 to 102.1%). In Stool sample positivity was 54.9% (396/779; 95% CI 41.0–68.8%), 45.2% (180/430; 95% CI 28.1–62.3%) and 34.7% (4/38; 95% CI − 29.5 to 98.9%) for SARS CoV-2, MERS CoV, and SARS CoV, respectively. In blood sample the positivity was 33.3% (7/21; 95% CI 13.2–53.5%), 23.7% (42/277; 95% CI 10.5–36.9%) and 2.5% (2/81; 95% CI 0.00–5.8%) for MERS CoV, SARS CoV-2 and SARS CoV respectively. SARS‐CoV‐2 along with previous two pandemic causing viruses from this family, were highly detected stool and saliva. A low positive rate was recorded in blood samples. Viruses were also detected in fluids along with unusual samples like semen and vaginal secretions thus highlighting unique pathogenic potential of SARS‐CoV‐2.

## Introduction

The human corona virus first identified in 1965 by Tyrrell and Bynoe^1^. These viruses were known to cause respiratory illness in hospitalised patient^2^. Coronavirus diseases 2019 (COVID‐19) is a highly infectious and an emerging respiratory disease caused by a Severe Acute Respiratory Syndrome Corona Virus-2 (SARS‐CoV‐2). With the progress of the pandemic new insights are coming into light about the virus. This is a third virus from this lineage to cause such a severe outbreak which has rapidly engulfed the whole world. Pandemics have been caused previously by viruses of this family like Middle East Respiratory Corona Virus (MERS CoV), Severe Acute Respiratory Syndrome Corona Virus (SARS CoV).

Although these viruses are primarily respiratory viruses, but they have been isolated from non-respiratory samples as well. Presently, the detection rate of SARS‐CoV‐2 RNA from different clinical specimens using Real Time Reverse Transcriptase Polymerized Chain Reaction (qRT‐PCR) after onset of symptoms is not yet well established. A recent study by Bwire et al. revealed a variable profile with a high detection rate of virus from lower respiratory tract (LRT) specimens, low detection from blood and zero detection from urogenital tract specimen, that is, urine^3^.

Therefore, the aim of this systematic review was to establish the profile of detecting SARS‐CoV‐2, MERS CoV, SARS CoV from different types of clinical specimens other than the respiratory specimen using a standard diagnostic test (qRT‐PCR).

## Material and methods

### Protocol developments

The protocol was based on the question “what is positivity rate for detecting human corona virus specifically SARS CoV, MERS CoV, SARSCoV-2 in different type of non-respiratory clinical specimen?”. We systematically reviewed the literature on SARS CoV, MERS CoV, SARS CoV-2, and its detection in non-respiratory samples by qRT-PCR technique. This review was designed on the basis of the Preferred Reporting Items for Systemic Reviews and Meta-Analyses Protocol (PRISMA) guideline^4^.

### Search strategy

A systematic search was conducted from database PubMed, Google scholar, science direct and Scopus. The data form key health care origination WHO and CDC was also searched. The search keywords in PubMed (MeSH terms and free text words) includes ‘COVID-19 and clinical samples’ ‘MERS and clinical samples’ ‘SARS and clinical samples’ google scholar search engine was searched with following key words ‘COVID-19 in urinary samples’ ‘COVID-19 stool samples’ ‘COVID-19 in semen samples’ ‘COVID-19 in vagina fluids’ ‘COVID-19 in peritoneal fluid’ ‘COVID-19 in blood’ ‘COVID-19 serum sample’ ‘COVID-19 in CSF’ ‘COVID-19 in pleural fluid’ ‘COVID-19 in pericardial fluid’ ‘COVID-19 in conjunctival sample’ ‘COVID-19 in amniotic fluid’ ‘COIVD-19 and pregnancy’ ‘COVID-19 in saliva’ ‘MERS in urinary samples’ ‘MERS stool samples’ ‘MERS in semen samples’ ‘MERS in vagina fluids’ ‘MERS in peritoneal fluid’ ‘MERS in blood’ ‘MERS serum sample’ ‘MERS in CSF’ ‘MERS in pleural fluid’ ‘MERS in pericardial fluid’ ‘MERS in conjunctival sample’ ‘MERS in amniotic fluid’ ‘MERS and pregnancy’ ‘MERS in saliva’ ‘SARS in urinary samples’ ‘SARS stool samples’ ‘SARS in semen samples’ ‘SARS in vagina fluids’ ‘SARS in peritoneal fluid’ ‘SARS in blood samples’ ‘ SARS serum sample’ ‘ SARS in CSF samples’ ‘SARS in pleural fluid’ ‘SARS in pericardial fluid’ ‘SARS in conjunctival sample’ ‘SARS in amniotic fluid’ ‘SARS and pregnancy’ ‘SARS in saliva’. SCOPUS database was also searched with similar key word for relevant articles. The data collected in timeline of 2003 to June 2020. Data was searched in between May 2020 to August 2020.

**Figure 1:**
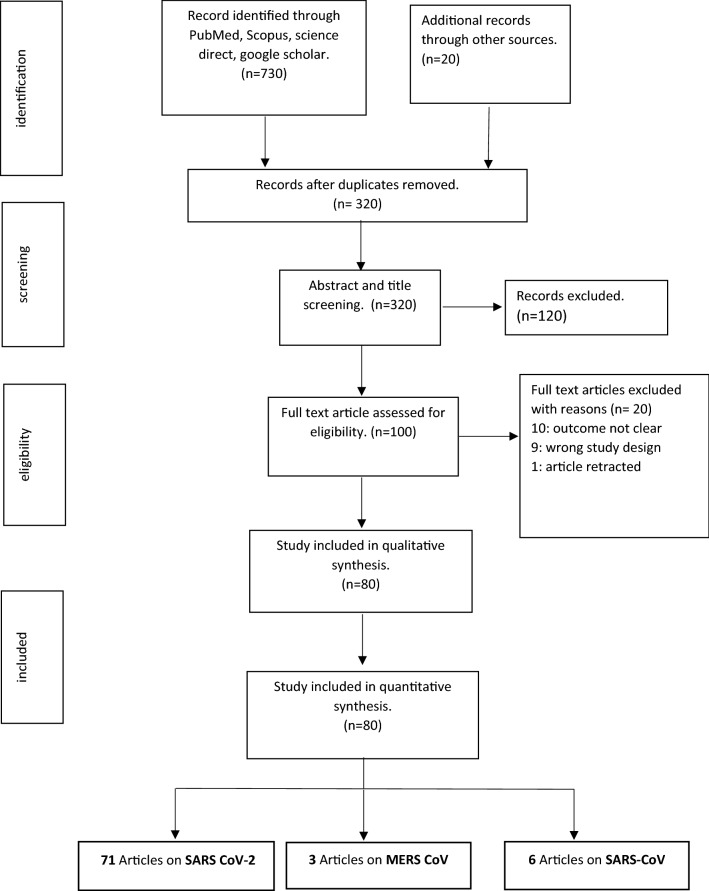
PRISMA flow chart showing study screening.

The search terms were combined using Boolean logic “or” and “and”. Filters were set to exclude non-human studies. Finally, the article that was directed to relevant categories as indicated in the PRISMA flow diagram (Fig. 1).

### Eligibility criteria

Clinical trial and observational studies including cross-sectional studies, retrospective studies and prospective studies were selected for review. Case series and case study including even one patient per study was considered. This review is limited and focused to isolation of the Human Corona virus specifically SARS CoV, MERS CoV, SARS CoV-2 by real time Reverse Transcriptase Polymerase Chain Reaction i.e., qRT-PCR. The studies that had targeted non-respiratory sample positivity for the qRT-PCR was taken in consideration. The studies which were conducted to determine diagnostic accuracy, reviews and non-human articles were excluded.

### Data extraction

The selection of the articles was done by 4 independent reviewers (SM, CM, JKM and ST) who evaluated the articles for the potential inclusion by screening the titles and abstract followed by full-text screening to determine eligibility of inclusion of the article for final study. Any discrepancies in inclusion of the articles were resolved by SM. The single specimen positive by qRT-PCR was taken into study. The number of patients tested were counted for each type of the specimen rather than the number of samples.

### Result analysis

Among 80 articles included the all the positive cases were added for the study. Information was recorded if there at least one specimen tested positive (confirmed cases). Different types of non-respiratory clinical samples were recorded for SARS CoV, MERS CoV, SARS CoV-2. Various type of clinical samples like stool/faecal, anal/rectal swab, urine, semen, testes tissue, vaginal secretions/mucus, vaginal swabs, placental specimen, amniotic fluid, whole blood, serum, plasma, cord blood, ascitic fluid, peritoneal fluid, gastric fluid, pericardial fluid, pleural fluid, breast milk, saliva, ocular surface samples, ocular swab sample, conjunctival swab, tears, CSF were taken in consideration. The rectal/anal swab specimen in the studies that also included stool/faecal specimen, the sample with maximum positivity was taken into account i.e. the stool/faecal specimen, in following studies by Huang et al.^5^, Wu et al.^6^, Robertson et al.^7^ and Liu et al.^8^.

The final analysis was made under following samples headings:- COVID19 stool sample (includes stool, faecal and anal/rectal swab sample), MERS stool sample, SARS stool sample; COVID19 urine sample, MERS urine sample, SARS urine sample; COVID19 semen sample; COVID19 vaginal sample (includes vaginal swabs sample, vaginal mucus sample, vaginal secretion sample); COVID19 placental sample (includes placental tissue specimen, post-delivery placental specimen, foetal and maternal placental specimen);SARS placental; COVID19 amniotic fluid sample; COVID19 blood sample, MERS blood sample, SARS CoV blood sample; COVID19 serum sample, MERS serum sample, SARS CoV serum sample; COVID19 plasma sample; COVID19 cord blood sample; SARS cord blood; COVID19 peritoneal sample; COVID19 pericardial sample; COVID19 breast milk sample; SARS breast milk sample; COVID19 saliva sample, SARS saliva sample; COVID19 ocular sample (includes conjunctival swab sample, ocular surface sample, tears sample, ocular swab sample), SARS ocular sample; COVID19 CSF sample, SARS CSF sample; COVID19 gastric fluid sample; COVID19 pleural fluid sample.

### Characteristic of the studies included

Out of 750 pooled studies 80 studies were chosen for the final analysis among which there were 34 Case report^7,9–41^, 15 Case series^5,8,42–54^, 14 Observational studies^55–68^,nine Cross-sectional studies^6,69–76^,three Retrospective Case series^77–79^, two Retrospective Cohort study^80,81^, two Cohort study^82,83^ and one Prospective interventional study^84^. Of 80 studies 50 were done in China. Among the 80 studies positive detection rate was extracted from all studies for all non-respiratory samples reported from that study. Data of respiratory samples was excluded from analysis only data of non-respiratory samples was included for analysis. Information was recorded if sample tested positive once irrespective of duration of illness. Samples included for analysis were have been specified in result analysis.

### Data synthesis

**Figure 2:**
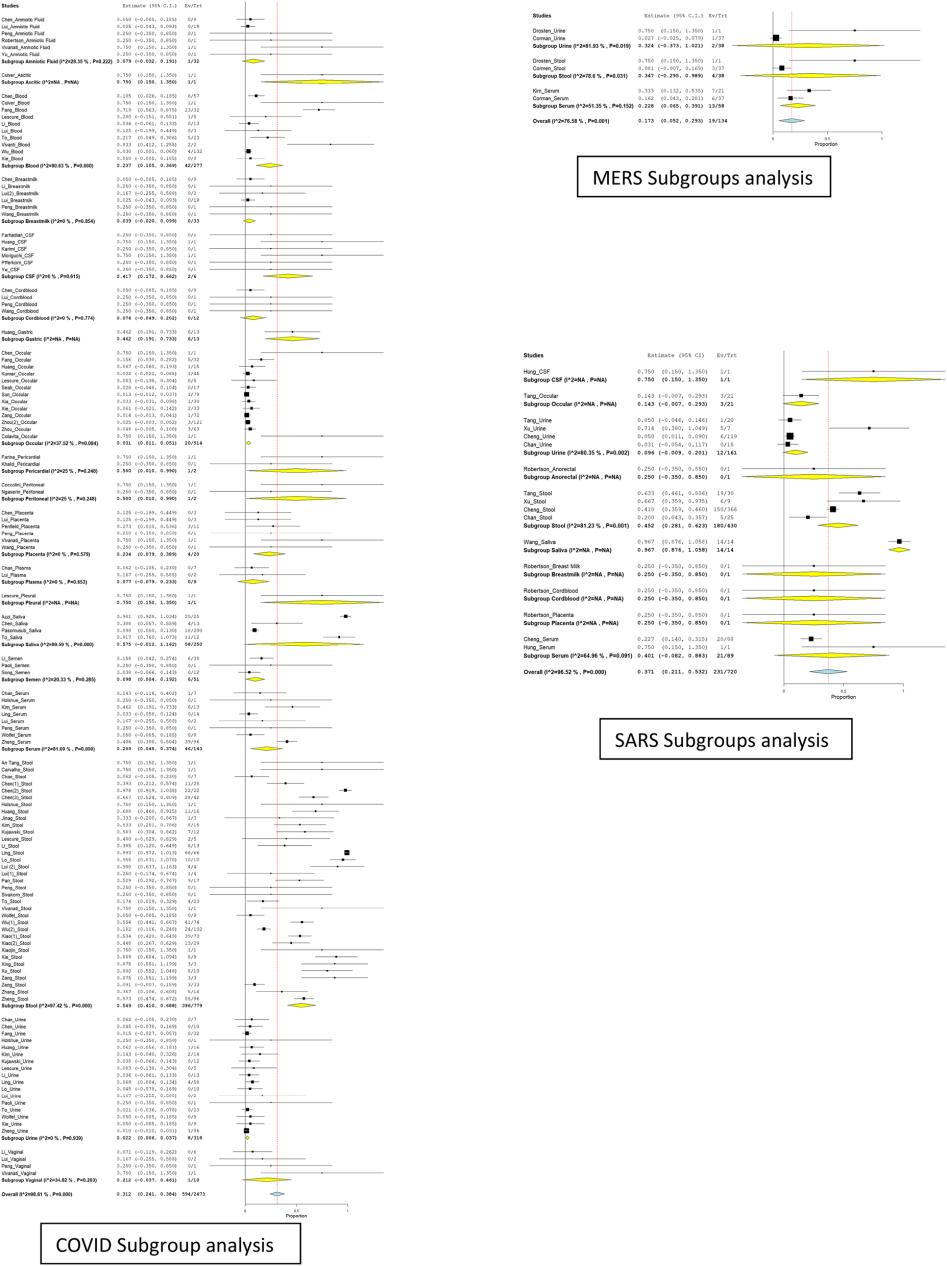
Forest plot of Sub group analysis for Heterogeneity in the analysed studies using Cochrane Q-statistic test and *I*^2^ statistic on different types of clinical specimens.

Collected data were verified and tabulated in Microsoft Excel software version 2009. Subsequently, Der Simonian–Laird (DL) binary random‐effects analysis was performed to establish a summary estimate (test positivity rate/proportion) by a random-effects model using Open Meta Analyst software^85^. The results were expressed using by pooled effect estimates and their 95% confidence intervals (CIs) and a correction factor of 0.5. Heterogeneity is commonly observed within diagnostic test accuracy reviews due to the differences in study quality, sample size, method, and different outcomes for which random effects models were kept default. Heterogeneity in the analysed studies was determined using Cochrane Q-statistic test and *I*^2^ statistic with subgrouping performed based on the types of clinical specimens. (Fig. 2). Between-study heterogeneity assessed using and *I*^2^ statistic. *I*^2^ ranges from 0 and 100% and larger values represent increasing heterogeneity (Table 1).

**Table 1:** Subgroup analysis (Specimen) and meta-regression for heterogeneity using Random-Effects Model.

COVID subgroups	Studies	OR (95% CI)	Q (df)	*I*^*2*^ (%)	Z value	S.E	P value	P value (het.)
Amniotic fluid	6	0.079 (− 0.032, 0.191)	6.978 (5)	28.35%	1.395	0.057	0.163	0.222
Ascitic	1	0.750 (0.150, 0.306)	NA	NA	NA	0.306	NA	NA
Blood	10	0.237 (0.105, 0.369)	96.028 (9)	90.63%	3.526	0.067	< 0.001	< 0.001
Breastmilk	6	0.039 (− 0.020, 0.099)	1.963 (5)	0%	1.295	0.030	0.195	0.854
CSF	6	0.417 (0.172, 0.662)	3.556 (5)	0%	3.333	0.125	< 0.001	0.615
Cordblood	4	0.076 (− 0.049, 0.202)	1.111 (3)	0%	1.190	0.064	0.234	0.774
Gastric	1	0.462 (0.191, 0.733)	NA	NA	NA	0.138	NA	NA
Occular	13	0.031 (0.011, 0.051)	13.584 (11)	19.02%	3.025	0.010	0.002	0.257
Pericardial	2	0.500 (0.010, 0.990)	1.333 (1)	25%	2.000	0.250	0.046	0.248
Peritoneal	2	0.500 (0.010, 0.990)	1.333 (1)	25%	2.000	0.250	0.046	0.248
Placenta	6	0.234 (0.079, 0.389)	3.798 (5)	0%	2.967	0.079	0.003	0.579
Plasma	2	0.077 (− 0.079, 0.233)	0.202 (1)	0%	0.965	0.080	0.335	0.653
Pleural	1	0.750 (0.150, 1.350)	NA	NA	NA	0.306	NA	NA
Saliva	4	0.575 (− 0.012, 1.162)	738.580 (3)	99.59%	1.921	0.299	0.055	< 0.001
Semen	3	0.098 (0.004, 0.192)	2.510 (2)	20.33%	2.044	0.048	0.041	0.285
Serum	8	0.209 (0.045, 0.374)	38.223 (7)	81.69%	2.501	0.084	0.012	< 0.001
Stool	35	0.549 (0.410, 0.688)	1316.023 (34)	97.42%	7.733	0.071	< 0.001	< 0.001
Urine	17	0.022 (0.006, 0.037)	8.326 (16)	0%	2.748	0.008	0.006	0.939
Vaginal	4	0.212 (− 0.037, 0.461)	4.603 (3)	34.82%	1.666	0.127	0.096	0.203
**MERS subgroups**								
Urine	2	0.324 (− 0.373, 1.021)	5.533 (1)	81.93%	0.911	0.356	0.362	0.019
Stool	2	0.347 (− 0.295,0.989)	4.672 (1)	78.6%	1.060	0.327	0.289	0.031
Serum	2	0.228 (0.065, 0.391)	2.056 (1)	51.35%	2.736	0.083	0.006	0.152
**SARS subgroups**								
CSF	1	0.750 (0.150, 1.350)	NA	NA	NA	0.306	NA	NA
Occular	1	0.143 (− 0.007, 0.293)	NA	NA	NA	0.076	NA	NA
Urine	4	0.096 (− 0.009, 0.201)	15.266 (3)	80.35%	1.794	0.054	0.073	0.002
Anorectal	1	0.250 (− 0.350, 0.850)	NA	NA	NA	0.306	NA	NA
Stool	4	0.452 (0.281, 0.623)	15.981 (3)	81.23%	5.174	0.087	< 0.01	0.001
Saliva	1	0.967 (0.876, 1.058)	NA	NA	NA	0.046	NA	NA
Breastmilk	1	0.250 (− 0.350, 0.850)	NA	NA	NA	0.306	NA	NA
Cordblood	1	0.250 (− 0.350, 0.850)	NA	NA	NA	0.306	NA	NA
Placenta	1	0.250 (− 0.350, 0.850)	NA	NA	NA	0.306	NA	NA
Serum	2	0.401 (− 0.082, 0.883)	2.854 (1)	64.96%	1.628	0.246	0.103	0.091

### Sensitivity analysis

**Figure 3:**
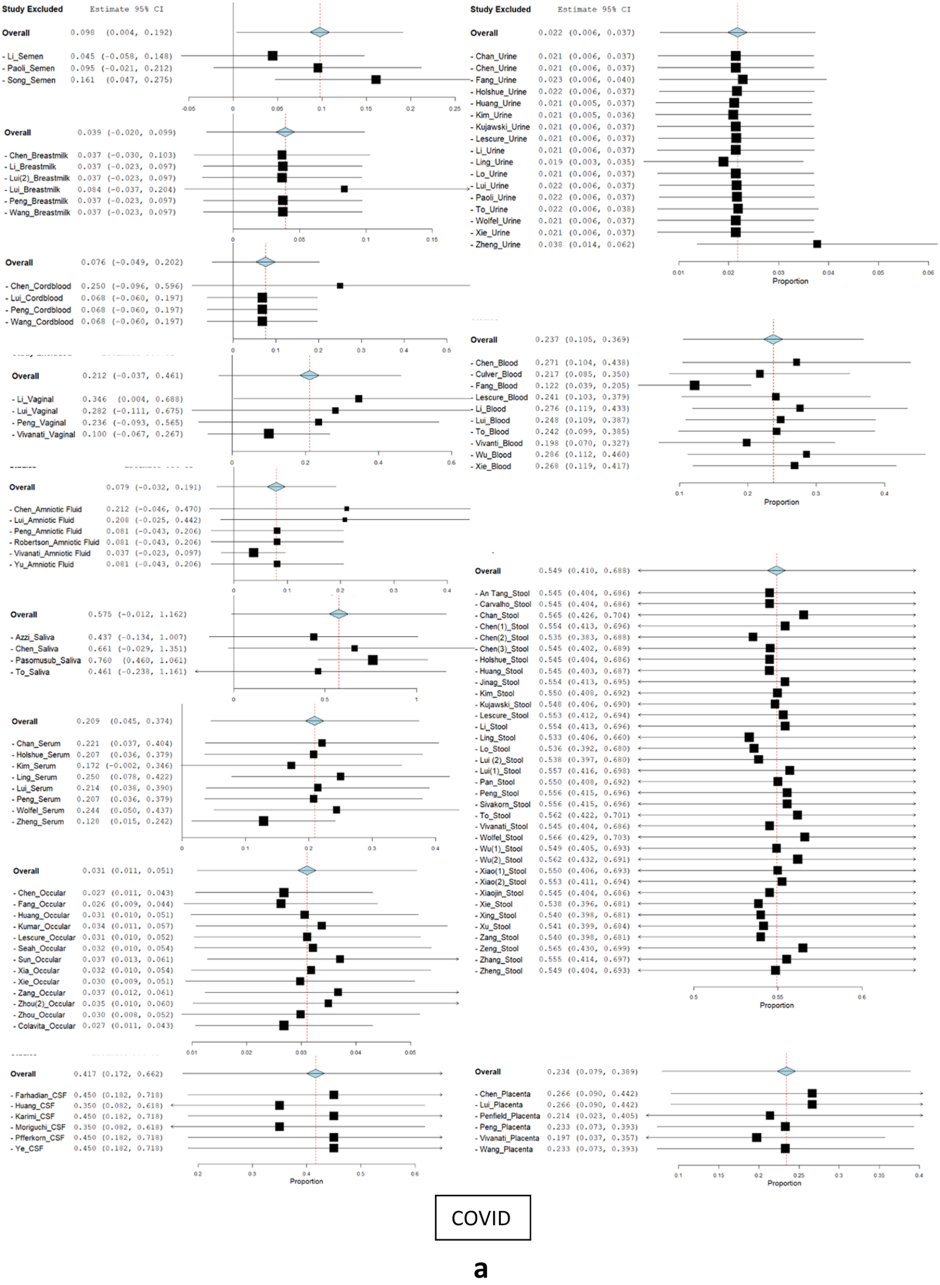

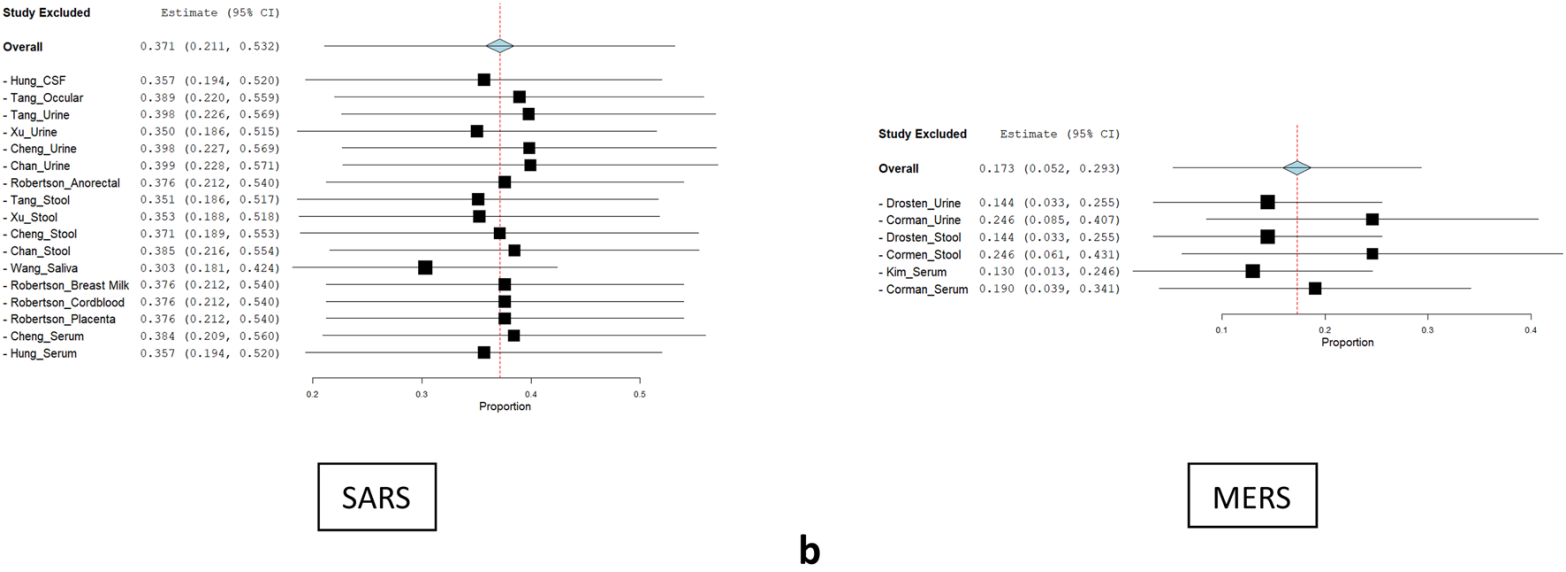
(**a**) Sensitivity analysis (leave one out) forest plot for COVID. (**b**) Sensitivity analysis (leave one out) forest plot for SARS and MERS.

Sensitivity analysis was done to determine the effect of individual studies on the pooled estimates. Univariate meta-regression was performed from primary studies using the random-effects model. A leave-one-out sensitivity analysis was done to assess heterogeneity between the study results. It was performed by iteratively removing one study at a time to confirm that our findings were not driven by any single study (Fig. 3a,b). Fewer studies in the group like: Culver et al. (Ascitic)^15^, Huang et al.(Gastric)^5^, Farina et al. & Khalid et al. (Pericardial)^21,38^, Coccolini et al. & Ngaserin et al. (Peritoneal)^39,59^, Lescure et al. (Pleural)^49^ weren’t applicable to the model.

### Quality assessment

**Figure 4:**
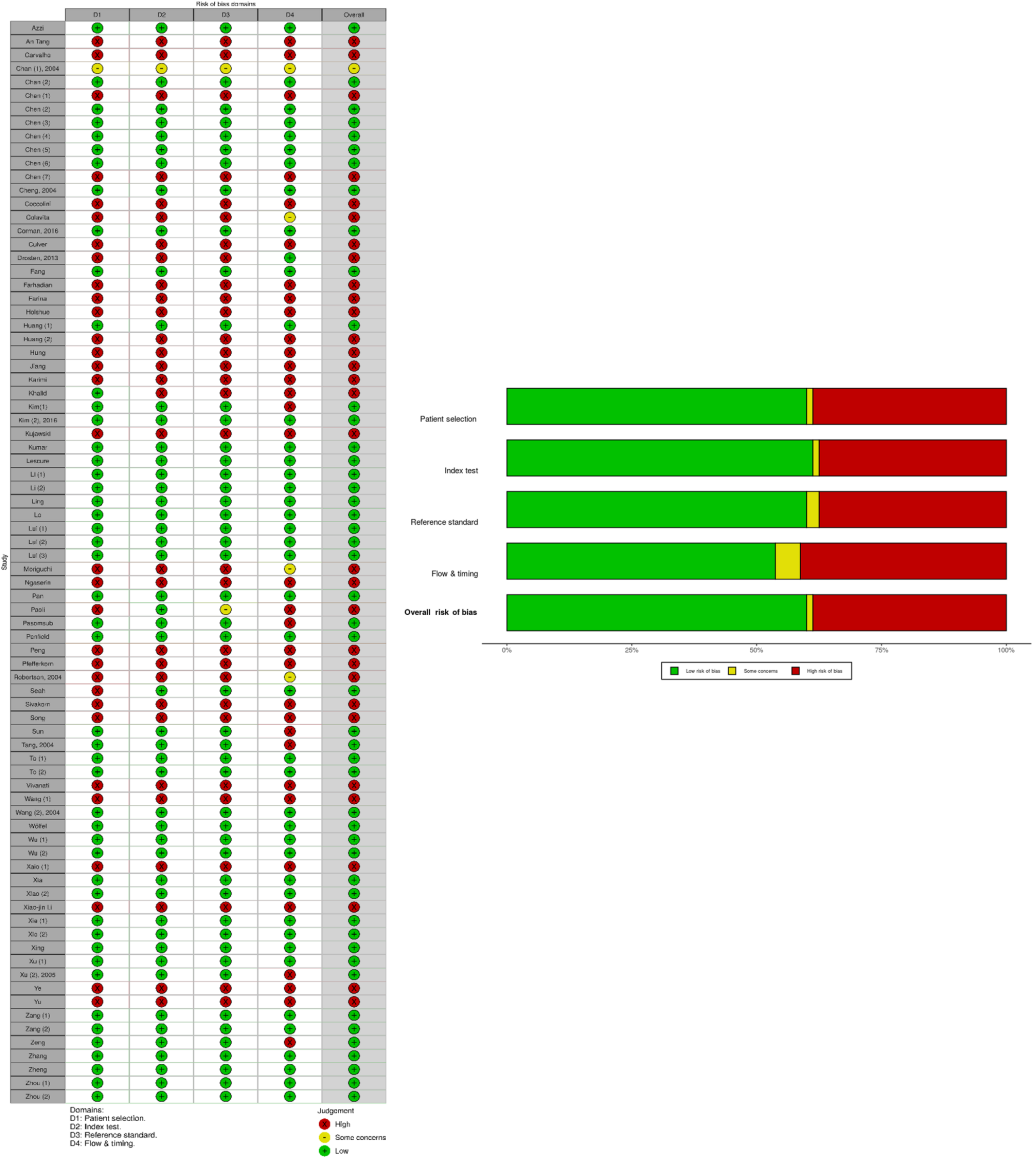
Risk of Bias assessment of included studies using QUADAS 2 tool displayed (traffic light plot) with bias summary (non-weighted).

The assessment was performed by four independent reviewers and further checked by two additional reviewers. As recommended by Cochrane Handbook for Systematic Reviews of Diagnostic Accuracy^86^, we adopted QUADAS-2 (Quality Assessment of Diagnostic Accuracy Studies -2)^87^ to evaluate the bias and quality of selected studies. The following four domains were considered for risks of bias and application concerns as depicted in the assessment tool: (1) participant selection; (2) index test; (3) reference text; and (4) flowing and timing. Studies with more than one “high risk of bias” were excluded (Fig. 4).

### Publication bias

**Figure 5:**
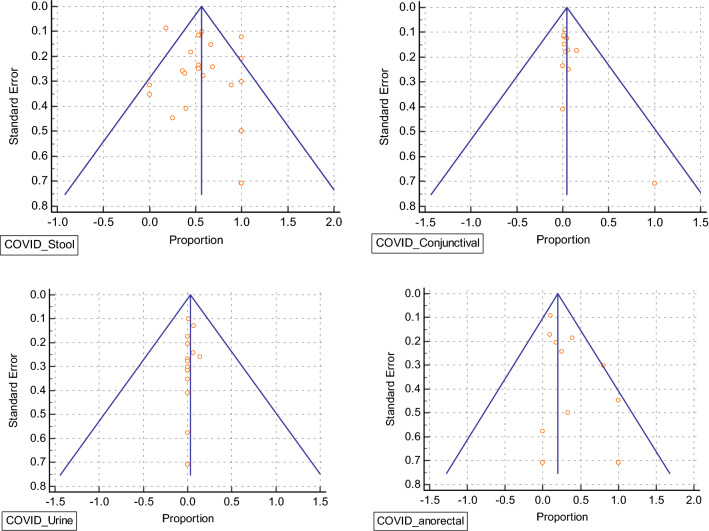
Funnel plot based on egger bias.

Output created by Open Meta Analyst software through a cumulative forest plot was indirectly used to assess the publication bias^88^. Funnel Plot and and Egger’s and Begg tests were conducted to assess the publication bias (P > 0.05) of the enclosed literature (Table 2). As seen in (Fig. 5), the funnel plot appearing symmetric, indicates absence of substantial publication bias.

**Table 2:** Funnel plot and and Egger’s and Begg tests were conducted to assess the publication bias of enclosed literature.

Study group	Egger’s test (Intercept, P value)	Begg’s Test (Kendall’s Tau, P value)	Publication Bias
COVID_stool	1.0166, 0.4720	0.05076, 0.7221	Absent
COVID_ occular	2.3073, 0.0055	0.5584, 0.0079	Present
COVID_urine	0.4802, 0.2650	0.4586, 0.0102	Absent
COVID_anorectal	2.1507, 0.0275	0.1622, 0.4401	Present

### Positive detection rate from non-respiratory samples

A total of 3429 non-respiratory specimens were recorded having SARS CoV (total sample–802), MERS CoV (total sample–155), SARS CoV-2 (total sample -2347). Here number of samples corresponds to number of patients. As we observed high rates of data heterogeneity further sensitivity analysis was undertaken to identify its potential source (study).

Out of all the samples studied high positive rate was seen for saliva with pooled detection rate of 96.7% (14/14; 95% CI 87.6–100.0%) for SARS CoV and 57.5%(58/250; 95% CI  − 1.2 to 116.2%) for SARS CoV-2, while low detection rate in urine samples for SARS CoV-2 with 2.2% (8/318; 95% CI 0.6–3.7%) and 9.6% (12/61; 95% CI − 0.9 to 20.1%) for SARS CoV but there was relatively higher positivity in urine samples for MERS CoV with detection rate of 32.4% (2/38; 95% CI − 37.3 to 102.1%).In Stool sample positivity was 54.9%(396/779; 95% CI 41.0–68.8%), 45.2%(180/430; 95% CI 28.1–62.3%) and 34.7%(4/38; 95% CI − 29.5 to 98.9%) for SARS CoV-2, MERS CoV and SARS CoV respectively. In blood sample the positivity was 33.3% (7/21; 95% CI 13.2–53.5%), 23.7% (42/277; 95% CI 10.5–36.9%) and 2.5% (2/81; 95% CI 0.00–5.8%) for MERS CoV, SARS CoV-2 and SARS CoV respectively. Different detection rate of the three pandemic coronaviruses from different non-respiratory samples are shown in Tables 3 and 4.

**Table 3:** Detection of SARS, MERS, SARS CoV-2 in different non-respiratory samples.

Sn. No	Type of specimen	SARSCoV2 + (n/N)	MERS + (n/N)	SARS + (n/N)
1	Stool	396/779	4/38	180/431
3	Urine	8/318	2/38	12/161
4	Semen	6/51		
5	Vaginal	1/10		
6	Placental	4/20		0/1
7	Amniotic	1/31		0/1
8	Blood	42/277	7/21	2/81
9	Serum	46/143	13/58	21/89
10	Plasma	0/9		
11	Cord blood	0/12		0/1
12	Ascitic	1/1		
13	Peritoneal	1/2		
14	Gastric	6/13		
15	Pericardial	1/2		
16	Pleural	1/1		
17	Breast milk	0/33		0/1
18	Saliva	58/250		14/14
19	Ocular	20/514		3/21
20	csf	2/6		1/1
	**Total**	**594/2472**	**26/155**	**233/803**

**Table 4:** Pooled positivity of SARS, MERS, SARS CoV-2 in different non-respiratory samples from highest to lowest.

SARS saliva	96.7% (14/14; 95% CI 87.6–100.0%)
COVID pleural	75.0% (1/1; 95% CI 15.0–100.0%)
SARS CSF	75.0% (1/1; 95% CI 15.0–100.0%)
COVID ASCITIC	75.0% (1/1; 95% CI 15.0–100.0%)
COVID saliva	57.5% (58/250; 95% CI –1.2–116.2%)
COVID stool	54.9% (396/779; 95% CI 41.0–68.8%)
COVID peritoneal	50.0% (1/2; 95% CI 1.0–99.0%)
COVID pericardial	50.0% (1/2; 95% CI 1.0–99.0%)
COVID gastric	46.2% (6/13; 95% CI 19.1–73.3%)
SARS stool	45.2% (180/430; 95% CI 28.1–62.3%)
COVID CSF	41.7% (2/6; 95% CI 17.2–66.2%)
SARS serum	40.1% (21/89; 95% CI –8.2–88.3%)
MERS stool	34.7% (4/38; 95% CI –29.5–98.9%)
MERS blood	33.3% (7/21; 95% CI 13.2–53.5%)
MERS urine	32.4% (2/38; 95% CI –37.3–102.1%)
SARS amniotic	25.0% (0/1; 95% CI 0.0–85.0%)
SARS breast milk	25.0% (0/1; 95% CI 0.0–85.0%)
SARS placental	25.0% (0/1; 95% CI:0.0%–85.0%)
SARS cord blood	25.0% (0/1; 95% CI 0.0–85.0%)
COVID blood	23.7% (42/277; 95% CI 10.5–36.9%)
COVID placental	23.4% (4/20; 95% CI 7.9–38.9%)
MERS serum	22.8% (13/58; 95% CI 6.5–39.1%)
COVID vaginal	21.2% (1/10; 95% CI: –3.7–46.1%)
COVID serum	20.9% (46/143; 95% CI 4.5–37.4%)
SARS occular	14.3% (3/21; 95% CI 0.00–29.3%)
COVID semen	9.8% (6/51; 95% CI 0.4–19.2%)
SARS urine	9.6% (12/61; 95% CI − 0.9 to 20.1%)
COVID amniotic	8.1% (1/31; 95% CI –4.3–20.6%)
COVID plasma	7.7% (0/9; 95% CI–7.9–23.3%)
COVID cord blood	7.6% (0/12; 95% CI –4.9–20.2%)
COVID breast milk	3.9% (0/33; 95% CI –2.0–9.9%)
COVID occular	3.1% (20/514; 95% CI 1.1–5.1%)
SARS blood	2.5% (2/81; 95% CI 0.00–5.8%)
COVID urine	2.2% (8/318; 95% CI 0.6–3.7%)

## Discussion

This systematic review and meta-analysis provide comprehensive data on detection of pandemic causing coronaviruses from non-respiratory samples. Our findings suggest that all the viruses are mainly respiratory, but they can cause multisystem involvement. They are detected from stool, urine, semen, testes tissue, vaginal secretions, placental specimen, amniotic fluid, whole blood, serum, plasma, cord blood, ascitic fluid, peritoneal fluid, gastric fluid, pericardial fluid, pleural fluid, breast milk, saliva, ocular specimen/tears and CSF. This finding is supported by several studies demonstrating detection of these viruses from various non-respiratory samples enlisted above. These findings show that in clinical practice non-respiratory samples are also important. SARSCoV-2 seems to have a strong predilection for the angiotensin two converting enzyme (ACE-2): the wide expression in different human tissues (as well as in the lung also in the intestine, testicle, kidney, etc.,) would also justify different theoretical modes of transmission of the virus in addition to respiratory route.

Out of all the samples studied maximum positivity was seen for saliva. This study revealed that saliva specimen had positivity of 57.5% for SARS CoV-2 detection and 96.7%for SARS CoV^40,48,68,71^. Saliva has been accepted as reliable non-invasive specimen with high sensitivity in comparison to NPS and throat swab^48,71^. Now that FDA approved kits are also available for diagnosis of covid-19 from saliva, this can be a safe alternative to respiratory samples^89^. Saliva has the advantage of being easiest to collect amongst all samples. Although positivity rate of saliva for SARS CoV-2 seems lower in our analysis as compared to previous study^3^. It could be explained by the sample size of the study by Pasomsub et al. 2020 was 200 out of which only 19 patients were positive for COVID-19 by NPS of which 18 tested positive for the saliva specimen causing rest to be outliers leading to decreased percentage of positivity. SARS CoV and SARS CoV-2 both has equally detected from saliva but there were no studies on detection of MERS.

The stool sample is well established for high sensitivity for the corona viruses^29^. This study also had same interpretation though the positivity rate for MERS CoV was lower than that of the other two viruses. Nevertheless, stool sample can be an alternate sample for diagnosis, but its clinical relevance and infectiousness needs to be further evaluated. Although several studies have shown that SARS CoV-2 is secreted more than a month in stool sample^10,13,47,58,61,81^.

All the three viruses have been detected from both serum and blood during early period of viremia^28,44,70^ It is highest for SARS-CoV-2 followed by MERS-CoV and SARS CoV. But the detection rate is highly variable depending on the timing of sample collection and significantly less as compared to respiratory sample. Nevertheless, it can complement diagnosis in absence of positivity from respiratory samples^90^.

Another interesting finding was detection of these viruses from genito-urinary specimen. There was 2.2% positivity in urine samples for SARS CoV-2,32.4%for MERS-CoV and 9.6%for SARS CoV. Detection of SARS CoV-2 from semen^9,33,82^ sample was 9.8% and vaginal^26^ swabs were 21.2%. Detection from these samples highlight unique pathogenic potential of this virus which needs to be evaluated further.

The body fluids like peritoneal^21,38^, ascitic, pleural, pericardial and CSF^14,16,20,31,34,35^ also had positive findings by qRT-PCR as seen in various studies. SARS CoV-2 was detected in ascitic fluid (1/1), in peritoneal fluid (1/2), in gastric fluid (6/13), pericardial (1/2), in pleural (1/1). CSF positivity was seen in two out of six patients for SARS CoV-2 and in one patient for SARS CoV.

The studies on ocular samples like tears and conjunctival swab samples positivity for the corona viruses was suggestive for ocular infection. Positivity in ocular samples was 3.1%for SARS CoV-2 and 14.3%for SARS CoV.

There were studies that arises question about vertical transmission of corona viruses. Positivity in placental samples23.4% and in amniotic fluid 8.1%for SARS CoV-2 was seen in post-delivery women, but none were found for the SARS CoV. No study for the MERS CoV was found. The post-delivery cord blood sample was not positive in SARS CoV-2 and SARS CoV. Similarly, none of the three corona viruses were not detected in the breast milk samples from pregnant woman.

To our knowledge, this is the first systematic review to comprehensively examine and compare SARS-CoV-2, SARS-CoV, and MERS-CoV detection in non-respiratory samples. Our study has limitations. First, almost all patients in the included had detection from non-respiratory samples at various time points which might have affected the results. Second, our meta-analysis identified substantial study heterogeneity, probably due to differences in study population, different extraction methods and kits used in RT-PCR. Thirdly, most of the studies published are case reports or case series where patient selection may have been biased. As a result, our analyses were based on varying methods, sample timing, sample frequencies and study endpoints differing widely between the included studies impairing contrasts and strong conclusions.

We recognized a systematic review and meta-analysis published on detection of SARS-CoV-2 from different clinical specimen using RT-PCR that included studies published up until may2020. The study include 7 studies and also analysed all samples including respiratory samples^3^. Our study on the other hand has analysed 80 studies wherein a total of 3429 samples were included. Besides, we have also compared the positivity in non-respiratory specimen of the three pandemic causing viruses which has not been attempted elsewhere.

This review provides detailed understanding about the evidence available so far on detection of SARS-CoV-2, SARS-CoV, and MERS-CoV from non-respiratory samples. It has implications in understanding viral dynamics and possible transmission routes to determine preventive steps that need to be taken. Various mitigation and prevention strategies for the current ongoing pandemic should be designed and implemented keeping in mind that this virus could have non-respiratory route of transmission.

## Conclusion

In this study, SARS‐CoV‐2 along with previous two pandemic causing viruses from this family, were highly detected stool and saliva. A low positive rate was recorded in blood samples. Also, viruses were also detected in fluids along with unusual samples like semen and vaginal secretions thus highlighting unique pathogenic potential of SARS‐CoV‐2. Thus, mitigation and prevention strategies should also focus on non-respiratory routes of transmission for the current pandemic.
